# T7 expression plasmids for producing a recombinant human G1P[8] rotavirus comprising RIX4414 sequences of the RV1 (Rotarix , GSK) vaccine strain

**DOI:** 10.1128/MRA.00603-23

**Published:** 2023-10-11

**Authors:** Asha A. Philip, Chantal A. Agbemabiese, Guanghui Yi, John T. Patton

**Affiliations:** 1 Department of Biology, Indiana University Bloomington, Bloomington, Indiana, USA; 2 Department of Electron Microscopy and Histopathology, Noguchi Memorial Institute for Medical Research, College of Health Sciences, University of Ghana, Accra, Ghana; Katholieke Universiteit Leuven, Leuven, Belgium

**Keywords:** rotavirus, reverse genetic analysis, vaccines, RIX4414 virus, recombinant virus

## Abstract

The live oral rotavirus RV1 (Rotarix) vaccine is formulated from the human G1P[8] RIX4414 virus. Based on RIX4414 sequences, T7 expression plasmids were constructed that supported recovery of recombinant RIX4414-like viruses by reverse genetics. These plasmids will advance the study of the RV1 vaccine, possibly allowing improvements to its efficacy.

## ANNOUNCEMENT

RV1 (Rotarix , GSK), the most widely used rotavirus vaccine, is formulated from the human G1P[8] virus RIX4414 (1, 2). To protect against rotavirus gastroenteritis, >24 million children received the RV1 vaccine in 2021 (2). A challenge to rotavirus immunization efforts is that RV1 and other rotavirus vaccines have vaccine efficacies in low-income countries (50%–64%) that can be significantly lower than in high- and moderate-income countries (85%–98%) (3, 4). We report here the construction of T7 plasmids that allow for the recovery of a recombinant (r) RIX4414-like virus by reverse genetics. By this method, it may be possible to generate modified forms of the RV1 vaccine with improved performance in low-income countries.

The rotavirus genome consists of 11 segments of double-stranded RNA (5). The rotavirus strain RIX4414 (originally named 89-12) was isolated from a child with acute gastroenteritis in 1989 and serially passaged in cell culture to promote the introduction of attenuating mutations (6). In this study, we designed 11 pT7 expression plasmids, each containing a cDNA sequence corresponding to one of the RIX4414 genome segments, using sequencing information for RIX4414 available in GenBank (Table 1). In cases where RIX4414 sequence information was missing, vis-a-vis, portions of the 5' and 3'-untranslated regions, sequence information for the prototypic human G1P[8] Wa virus was used instead (Table 1). Because the original pT7/RIX4414 VP2 plasmid was not functional in the reverse genetics system, the VP2 coding region was slightly modified to include residues common to other human G1/4P[8] virus strains [e.g., Wa, KU (7), Odelia (8)] (Table 1). To generate pT7/RIX4414 plasmids, RIX4414 cDNA sequences were made with an upstream T7 promoter and downstream hepatitis D virus ribozyme and T7 terminator, by Gibson assembly of oligonucleotide fragments (9, 10). These cDNAs were inserted into the *EcoRV* site of pUC-GW-Amp plasmids (Azenta).

**TABLE 1:** RIX4414 sequences used in the generation of T7 expression plasmids

Genome segment	RIX4414 sequence reported in GenBank (RVA/Human-lab/ USA/ Rotarix_SSCRTV_00 092/2016 /G1P[X])	Source of RIX4414 sequences assembled into T7 expression plasmids^*a*^			Accession number		Percent sequence identity (nt/aa), T7 RIX4414 plasmid versus RIX4414 vaccine lab-strain
		Full/partial sequence	5′ UTR completed with RVA/Human-tc/USA/Wa/1974 /G1P[8]	3′ UTR completed with RVA/Human-tc/USA/Wa/1974 /G1P[8] or other genotype 1 strain	RIX4414 sequences in T7 expression plasmids	Vaccine RIX4414 (lab-strain)	
VP1	MF469220	MF469220 (full)			OR187571	OR187592	100.00/100.00
VP2	MF469221	MF469221 (full, with modifications S12N, N30D, P108S, K120R, I390T, V616T)^*b*^			OR187573	OR187593	99.78/99.32
VP3	MF469222	MF469222 (partial)	JX406749 (Wa)		OR187574	OR187594	99.96/100.00
VP4	JN849113	JN849113 (full)			OR187575	OR187595	99.92/99.75
VP6	MF469223	MF469223 (full)			OR187576	OR187596	99.93/100.00
VP7	MF469224	MF469224 (full)			OR187577	OR187597	99.91/100.00
NSP1	MF469215	MF469215 (partial)	JX406751 (Wa)	JX406751 (Wa)	OR187578	OR187598	99.87/100.00
NSP2	MF469216	MF469216 (partial)	JX406754 (Wa)	LC438386/LC485138 (KU/Odelia)	OR187579	OR187599	99.91/100.00
NSP3	MF469217	MF469217 (partial)		JX406753 (Wa)	OR187580	OR187600	99.91/100.00
NSP4	MF469218	MF469218 (partial)	JX406756 (Wa)	JX406756 (Wa)	OR187581	OR187601	100.00/100.00
NSP5	MF469219	MF469219 (full)			OR187582	OR187602	99.85/100.00

^*a*^ Full, T7 expression plasmid contains a complete cDNA sequence for a RIX4414 genome segment matching the GenBank number. Partial, complete rotavirus cDNA sequence in T7 expression plasmid was generated by combining the partial RIX4414 segment sequence reported by the GenBank number with the 5' and/or 3' UTR sequences of other Wa-like rotaviruses.

^*b*^ VP2 protein encoded by T7 expression plasmid was engineered to contain residues common to other genotype C1 VP2 segments previously used in developing reverse genetics systems (7, 8).

**Fig 1:**
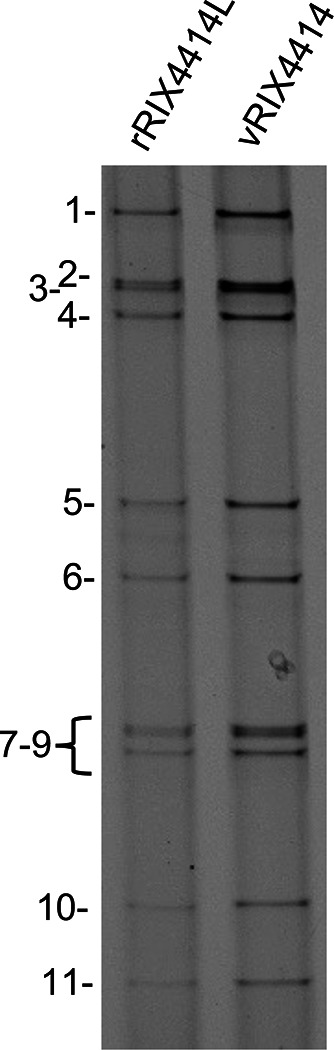
Recovery of recombinant RIX4414-like virus by reverse genetics. rRIX4414L and RIX4414 derived from RV1 vaccine (vRIX4414) were resolved by electrophoresis on a 10% polyacrylamide gel and stained with ethidium bromide. Genome segments 1–11 of rRIX4414L are indicated.

The pT7/RIX4414 plasmids supported recovery of rRIX4414-like virus using a modified reverse genetics procedure (11, 12). Briefly, BHK-T7 monolayers in 12-well plates were transfected with plasmid mixtures containing 0.8 µg of each pT7/RIX4414 plasmid (except the NSP2 and NSP5 plasmids, which were 4.8 µg each), 1.6 µg of pCMV/NP868R capping plasmid, and 1.6 µg of pcDNA/T7 RNA polymerase plasmid. Two days later, the transfected cells were overseeded with 10^5^ MA104 cells. Three days later, the BHK-T7/MA104 cells was overseeded with 10^5^ Vero cells. Eight days later, viruses in the cell lysates were amplified on Vero cells and plaque isolated on MA104 cells (13). The RNA genome of the rRIX4414-like isolate is shown in Fig. 1, and the genome sequence of the rRIX4414-like isolate was confirmed by Nanopore sequencing (14). Based on sequence analysis, the rRIX4414-like virus has nucleotide and amino acid sequence identities of >99% with the vaccine RIX4414 (lab-strain) virus (accession numbers for the lab strain provided in Table 1), making the rRIX4414 reverse genetics system a possible tool for investigating genetic changes that may improve RV1 performance.

## Data Availability

The pT7/RIX4414 plasmids will be provided upon request to the corresponding author. GenBank accession numbers for the pT7 plasmids are given in Table 1.
